# Draft Genome Sequence of *Salmonella enterica* subsp. *enterica* Serovar Napoli Strain SN310, Cause of a Multischool Outbreak in Milan, Italy, in 2014

**DOI:** 10.1128/genomeA.01044-15

**Published:** 2015-09-10

**Authors:** Pol Huedo, Maria Gori, Erika Scaltriti, Marina Morganti, Gabriele Casadei, Ettore Amato, Mirella Pontello

**Affiliations:** a Dipartimento di Scienze della Salute, Università degli Studi di Milano, Milano, Italy; b Istituto Zooprofilattico Sperimentale della Lombardia e dell'Emilia Romagna (IZSLER), Sezione di Parma, Parma, Italy

## Abstract

We report the draft genome sequence of *Salmonella enterica* subsp. *enterica* serovar Napoli strain SN310, isolated from a stool sample of an affected pupil during a multischool outbreak in 2014 in Milan, Italy. This represents the first reported draft genome sequence of the emerging serovar Napoli.

## GENOME ANNOUNCEMENT

In Europe, human infections caused by *Salmonella enterica* subsp. *enterica* serovar Napoli have increased by 256% since the beginning of the century, mainly affecting France, Switzerland, and Italy (1, 2). In the latter, a 28.2% incremental increase of this serotype has been observed in the past few years, and it has become the fifth most common serovar isolated from humans (3), with most cases being reported in northern regions. Particularly, in Lombardy *Salmonella* Napoli incidence (1.49 × 100,000 inhabitants) has recently exceeded that of *Salmonella enteritidis* (1.19 × 100,000 inhabitants), so it now ranks as the third most prevalent serotype, behind serovars 1.4.[5].12:i: and Typhimurium (6.98 and 2.66 × 100,000 inhabitants, respectively) (Italian surveillance system IT-ENTER-NET, 2014). Furthermore, in 2014, the largest *Salmonella* Napoli outbreak ever reported in Italy occurred in 4 primary schools in the Milan district, affecting 47 persons with 6 cases of invasive salmonellosis (12.8%). Here we report the draft genome sequence of the outbreak-related strain SN310, which represents the first reported draft genome sequence of *Salmonella* Napoli.

During our recent outbreak investigations, *Salmonella* Napoli strain SN310 was isolated from a stool sample of an affected pupil according to standard procedures. Serotyping was performed according to the White-Kauffmann-Le Minor scheme (4). Whole DNA was extracted using the GeneAll exgene tissue SV kit and underwent quality controls. Sequencing was performed on the Illumina MiSeq platform with a 2 × 250 paired-end run, after library preparation with a Nextera XT sample preparation kit (Illumina), which generated a total of 3,606,256 paired sequences. Reads were analyzed and quality checked using FastQC. Genome assembly was performed using Mira 4.0.4, resulting in 210 large contigs (length >1,000 bp) out of a total of 359, with an average coverage of 138.46×, for a total of 4,631,315 bp with a G+C content of 53%.

Genome annotation was performed by the NCBI Prokaryotic Genome Annotation Pipeline version 2.10 and 4,546 genes were predicted, of which there are 4,316 coding genes (CDS), 102 pseudogenes, 22 rRNAs, 80 tRNAs, and 26 noncoding RNAs (ncRNAs). *In silico* multilocus sequence typing (MLST) analysis revealed that SN310 belongs to the sequence type (ST) 474 and to the ST complex 60, STs previously identified among other *Salmonella* Napoli human strains isolated in France and Italy (5). Further PHAST analysis (6) identified 10 prophage regions, of which 1 region is intact, 7 regions are incomplete, and 2 regions are questionable. This new genome provides valuable information to understand *Salmonella* Napoli phylogeny as well as to identify the diverse virulence factors exhibited by this serovar.

### Nucleotide sequence accession numbers. 

This whole-genome shotgun project has been deposited at DDBJ/EMBL/GenBank under the accession number LFIH00000000. The version described in this paper is version LFIH00000000.1.
